# Chloroform as a Surgical Anesthetic, and Its Administration

**Published:** 1903-03

**Authors:** F. Manfred Call

**Affiliations:** Richmond, Va., Adjunct Professor Practice of Medicine, Medical College of Virginia


### CHLOROFORM AS A SURGICAL ANESTHETIC, AND ITS ADMINISTRATION.*

By F. MANFRED CALL, M.D., RICHMOND, VA.,
Adjunct Professor Practice of Medicine, Medical College of Virginia.

A study of this subject involves, primarily, three divisions: the anesthetist, the drug, and its administration, the whole representing the welfare of the patient.

As to the first, the anesthetist, it is well said that "No person who has not a wholesome fear of anesthetics can be trusted to administer them. In many cases the anesthetization transcends the operation in gravity and importance, and, to insure success, it is essential that one of skill and experience should conduct the administration."

He alone is responsible, and should have the sole direction of this most important function. Suggestions from surgeon, assistants or bystanders serve but to confuse and directly interfere with a successful and smooth narcosis. One who needs watching and close supervision clearly has no right to assume the grave responsibilities associated with this task, one which I am sorry to say is often left, the country over, for the latest arrival on the house staff, or used as a means to side-track a troublesome visitor.

One idea should predominate, first and foremost, rigid attention to the immediate business in hand; give the anesthetic, and leave the operation, its progress and interest, exclusively to the surgeon. How often have we seen the one at the head with eyes fixed on the field of operation in all-absorbing interest, the patient left to a too kind providence, and only recalled to his duty by an emergence from narcosis, or the rapid supervention of some alarming symptom necessitating the most active measures for resuscitation. In my experience most of the symptoms that develop ofttimes so unexpectedly have done so at a moment when my vigilance has been relaxed. I am convinced that a careful observer can in a measure anticipate, and, to a large extent, diminish their danger.

* Read before the Richmond Academy of Medicine and Surgery, February 10, 1903.

The logical conclusion affording the solution of the difficulty is the appointment of an expert anesthetist, certainly in the larger hospitals, and whenever possible the assistance of such a one in private cases. Unlike the junior members of the house staff, he does not wish to learn surgery; the field of operation has not an overpowering attraction for his mind and eye; he has a reputation to foster; the full burden of responsibility rests on his shoulders, and from past experience he has learned the many pitfalls that might entrap the unwary.

*Physiological Action.*—On the respiratory mucous membrane the vapor of chloroform is irritating, but much less so than that of ether. The respirations in the beginning of narcosis are deeper than normal, later becoming more rapid and shallow.

When absorbed, chloroform is not simply in solution, but is combined with the cholesterin and lecithin of the red corpuscles, with a reduction in the hemoglobin and consequent impairment of the oxygen-carrying power of the blood, and a reduction of the oxidation processes throughout the body. Mikulicz states that the administration of chloroform may reduce the amount of hemoglobin as much as five or ten per cent. and gives thirty per cent. hemoglobin as the minimum for the administration of this drug. Fish asserts that chloroform extracts oxygen from the oxyhemoglobin, combining with the hemoglobin to form a more stable compound. Given a hemoglobin percentage of fifty, the corpuscles cannot meet the physiological requirements of the body.

On the central nervous system it is depressant, in order, to the brain, cord and finally the medulla, with the sensory functions affected before the motor. Local nerve mechanism is affected as well.

Blood-pressure is lowered. Ordinarily this would stimulate the vasomotor center, but, for the automatic regulation of the circulation an unimpaired sensibility of this center is necessary; this we have seen is not intact, for the drug has already depressed the central, the local nerve mechanism, and the heart itself. There is a weakness in the auricular contractions, an increase in the ventricular relaxation, the heart coming to a standstill in diastole. In rapid chloroformization the sudden impact of the irritating vapor upon the peripheral nerves of the breathing surface may cause a contraction of the pulmonary arterial vessels, with an ischemia of the lungs and overpowering of the right heart.

Primary dilation of the pupil occurs, produced by reflex inhibition of the third nerve center; this is followed by a contracted pupil, due to complete subjection of the cerebrum, leaving an unopposed third nerve. In dangerous narcosis the third nerve center is poisoned and no longer controls the pupil, which, dilated, is less sensitive to light, with fixation of the globe (Ward).

Fatty changes are induced in the liver, kidneys, heart, etc., by the effect on the blood-cells and by direct effect on the tissue cells. The urine is so loaded with alkaloidal bodies that the kidneys cannot perform elimination with sufficient rapidity, resulting in a condition of autointoxication. Tests as to the permeability of the normal kidney before and after chloroformization show a ratio of 35–41, with reduced quantity of urine and increased acidity. When albuminuria exists prior to anesthesia, the amount may be increased; when non-existent, fifteen to twenty per cent. of cases may develop this symptom, the changes varying from hyperemia and capillary hemorrhage to extensive coagulation necrosis of renal epithelium (K. Ajello). Chloroform, by volume, is more irritating to kidney cells than ether, and its resulting nephritis is more likely to become chronic.

After death it is found that the reaction of the fluids and tissues of the body is decidedly acid.

Body temperature is reduced through increased output of heat by the skin and lessened heat production. Watson notes fall of temperature after any general anesthesia, more marked with chloroform, sometimes requiring twenty-four hours to regain normal. After reduction of body temperature, patients are more susceptible to microbic infection.

Elimination is accomplished mainly by the lungs; the drug is found also in the urine and the stomach, and is said to occur in the perspiration and the milk.

Ordinarily chloroform tends to decompose with the production of $HCl$ and carbonyl chloride, the latter causing the majority of cases of after-sickness. When administered by gaslight, its vapors are broken up into chlorine and carbonic oxide and produce bronchial irritation, with the possibility of asphyxia.

From this brief résumé of its physiological action, we may deduce the indications and contraindications to its use, and the special dangers.

Palmer gives the following indications for its choice :

1. Extensive bronchitis, pneumonia or other inflammatory conditions of the respiratory tract.

2. Acute and chronic nephritis.

3. Aneurism, degeneration of the vessel walls, high tension pulse.

4. Brain surgery.

5. Abdominal and pelvic operations.

To these may be added operations about the upper air-passages where the actual cautery is to be used. With foreign bodies in the larynx and trachea, chloroform anesthetizes more readily with less secretion of mucus, but with greater tendency to respiratory failure and reflex inhibitory phenomena (Crile).

Chloroform is contraindicated in fatty degeneration and dilatation of the heart, emphysema, extreme prostration, shock, collapse, and hemorrhage.

"Those of the so-called lymphatic temperament, neurasthenics, chlorotics, anemics, leukemics, withstand general anesthetization badly."

Valvular lesions increase danger only if they are obstructive, and even in such cases compensatory hypertrophy may suffice for the increased resistance encountered.

The type of chloroform blood-pressure curve shows subnormal pressure in ninety per cent. of all cases. Increased blood-pressure is found in a large number of cases in individuals past fifty years, while the instances of greatest diminution of blood-pressure are exclusively represented in children under fifteen. This is contradictory to the popular belief that children bear chloroform well. My experience confirms the truth of this statement.

During the administration, special dangers arise at certain stages of narcosis; early in inhalation from sudden paralysis of the cardiac ganglia, due to reflex action from peripheral irritation of an associated nerve; in the stage of rigidity from tetanic fixation of the respiratory muscles, with resulting venous stasis and cardiac cessation ; or there may be a paralytic relaxation followed by deep inspirations of surcharged air paralyzing cardiac or respiratory motor ganglia.

Crile has demonstrated a point of some interest. He has shown that manipulation of the brachial plexus, or of the nerves supplying the respiratory muscles, cause, by mechanical stimulation, an increased respiratory action. This is likely to signify to the anesthetist a condition of under anesthesia, while there is actually the danger of over anesthesia by an increased and excessive inhalation. Depression is likely to occur in proportion to the severity of the manipulation and the suddenness of its cessation, affecting most markedly the circulation and then the respiration.

Vomiting may occur in the early stages from a nauseating effect of the vapor or reflexly from irritation of the pharynx, and, later, during emergence from narcosis.

Cyanosis is frequent in short-necked individuals and drunkards. It is induced by unfavorable posture, follows cough and vomiting and may occur during the stage of rigidity, and from mechanical obstruction to respiration by a falling back of the tongue and epiglottis.

Before commencing the administration it is well to have a history of each case and a special examination made by the anesthetist of the mouth, nose, respiratory, and cardiac functions, a complete urinalysis, and, if necessary, blood examinations, together with an account of previous anesthetizations.

In emergency cases, and in intestinal obstruction with stercoraceous vomiting, gastric lavage is indispensable, as it empties the stomach, lessening the vomiting and the danger of the aspiration of particles of the gastric contents into the respiratory tract. In deliberate operations, time is given for a proper supervision and correction of all the emunctories, pulmonary, cutaneous, renal and alimentary. The bowels should be moved the night before, an enema given in the morning and catheterization just before going to anesthetizing room. On the morning of operation, no food of a solid character should be taken ; in patients weak or debilitated a glass of warm milk or tea should be given, or a nutrient enema, five or six hours before the operation. A nurse should always be present with a female patient, and in all cases an orderly or other assistant in case the patient prove obstreperous. Tying a patient in the beginning necessarily alarms and excites him. Within convenient reach, place hypodermic syringe with stimulants, a tank of oxygen and tongue forceps. As a routine measure, I generally precede the anesthetic with atropin gr. 1-100. This stimulates the respiratory functions, prevents cardiac inhibition from irritation of the vagus, checks excessive secretion in the upper air-passages and prevents the tendency to edema of the lungs by stimulation of the vasomotor center. It tends to raise the temperature of the body and increases rather than lessens the urinary secretion, and, in my opinion, facilitates, particularly when used as a part of the after-treatment, free movement of the bowels. The only objection is its action of obscuring the pupil symptoms.

Before and during the operation the posture should be as comfortable as possible, allowing free expansion of the chest and movement of the abdomen. There should be no high pillow under the head, no constrictions about the body, no foreign body in the mouth, and, when on the operating-table, the hands should be secured in such a position as will minimize the danger of post-operative paralysis. Reassure the patient and allow no conversation with bystanders or assistants. Note the normal size of the pupil. To prevent irritation from the vapor, protect the lips and nose with cold cream or vaseline and cover the eyes with a towel. I prefer the Esmarch's inhaler with a small pledget of cotton at the top; the cover to this cone should be sterile, thus avoiding the danger of infection of the air-passages. Put on a few drops of chloroform and hold the mask about three inches from the face, gradually bringing nearer as the patient becomes accustomed to the odor and is more composed, until, finally, the mask rests on the face, encircling the mouth and nose. Let the breathing be natural rather than abnormally deep, for very little vapor can be tolerated at first, and, if deep inspirations be given at the start, exhaustion and shallow breathing come on at a time when more of the vapor should be inhaled. Children can be told to blow away the cotton; older people, when drowsiness and slowed breathing begin, can count. The frequent addition of small quantities is far preferable to larger quantities at longer intervals. If the breathing can be kept regular, there is no danger in pushing the drug until complete narcosis. The degree of narcosis and danger is not due to the amount but to the concentration of the vapor, and this should never exceed four per cent. (Finney). As an index to regularity of breathing it is unsafe to depend entirely on the respiratory movement of the chest and abdomen, for this is often kept up even in the presence of a dangerous degree of cyanosis, as shown by inspection of the lips and face and altered character of the pulse.

The vapor at first irritates, occasionally provokes coughing, pupils dilate, pulse ordinarily becomes more frequent than normal, but slows down in very nervous subjects and may possibly go below seventy. Should struggling occur, do not push the anesthetic, for then deep breathing is induced, and if the cone is saturated a dangerous dose may be given. In many cases this is followed by a stage of rigidity with slow and shallow breathing, some cyanosis; finally relaxation occurs and the breathing becomes deeper and more regular, the muscles flaccid and complete narcosis rapidly supervenes.

As to the pupillary changes, there is primarily a slight dilatation, then contraction with reaction to light, until complete narcosis, when the minimum size is attained, with reaction to light and conjunctival reflex lost. Should the pupil now dilate, it is from one or two causes; the patient has either too much or too little of the drug; if the former, there is no conjunctival reflex, no reaction to light, but a climax of lowered blood-pressure with cardiac and respiratory failure. Should the latter be the case, the conjunctival reflex returns; the pupil reacts to light, relaxation of muscles begins to disappear, and, if the jaw be held forward as mentioned later, this is done with more effort on account of the rigidity of the muscles of the jaw and neck. One of the first and most easily observed symptoms of returning consciousness is rolling of the eyeballs. Operation should be begun only when the patient is thoroughly anesthetized, thereby preventing greater vasomotor shock and more prolonged manipulations. The preliminary scrubbing, placing of sterile towels, etc., may be done just before the stage of complete narcosis.

*Treatment of Complications, Preventive and Active.*—To insure regular breathing, it is essential that there be no obstruction to respiration, the most common cause being the falling back of the tongue and epiglottis. A point of great utility, which I have practiced for quite awhile, is so well described by Simpson, I will quote his words. It is in regard to the management of the lower jaw. "Place the thumb of the hand on the bridge of the nose at its root, palm applied to cheek; middle of ring finger presses from behind forward upon the posterior surface of the angle of the jaw, and, as the relaxation of the muscles occurs with approaching narcosis, the lower teeth are hooked in front of the upper." Fenger prevents obstruction from falling back of the tongue by making traction of the hyoid bone, passing a ligature around it for this purpose. In those cases where traction on the tongue becomes necessary, remember that when forcibly done there is danger of inhibition of respiratory and cardiac action, varying from slowed action to complete cessation. The same result follows too forcible extension of the head. Atropine prevents the cardiac but not the respiratory phenomena.

Much can be done for the prevention of collapse by a well-warmed room, proper and sufficient covering for the patient, hot water bags, dry table, etc. In prolonged operations it is well to suspend the administration of the vapor for a few minutes, letting the patient inhale oxygen of pure atmosphere, stopping short of consciousness. This procedure, in a measure, tends to regenerate the blood and prevent collapse.

Should collapse develop, withdraw the anesthetic, lower the patient's head but do not forcibly extend it ; if necessary, invert him, make gentle and rhythmic traction of the tongue (eighteen to the minute), perform artificial respiration with the first movement one of expiration, give inhalations of oxygen and hypodermic administration of strychnine gr. 1-30, atropine gr. 1-100, digitalin 1-100, caffeine, as necessary; normal saline solution may be poured into the abdominal cavity if the operation be a laparotomy, by hypodermoclysis if the circulation is sufficiently active to absorb it, or quicker still intravenous injection. In abdominal cases where the Trendelenburg position is used, there is less liability to syncope. Bandaging the lower extremities is often of service.

Do not wait to stimulate until the circulation is so poor that rapid absorption is difficult, but try to forestall by early application; thus, during the progress of an operation, if the patient is not progressing satisfactorily, and any manipulations increasing the shock or prolonging the operation are demanded, throw in the stimulant, and if the response is not decided, change, in the absence of greater contraindications, to ether.

A report of revival of the heart in an abdominal case by grasping the organ through the diaphragm and making rhythmical compression is given by Starling. Deep compression of the precordial region at the rate of 120 to the minute was successfully used by Green on a patient in whom, for a period of seven minutes, no heart beat or respiratory effort could be detected.

Suprarenal extract has a marked stimulating, though evanescent, effect upon respiratory action, the vasomotor system and the heart. The best method of administering is the intravenous injection of a one per cent. solution, using from fifteen to thirty grains.

The value of the faradic current is due more to its stimulating effect on respiration than to any special direct effect on the heart. Its use is attended with the danger of cardiac inhibition. A much milder current is required for the respiratory than for the cardiac stimulation.

One of the most frequent and distressing of the sequelæ is the after vomiting. The most important preventive measure is the preliminary attention to the alimentary tract. I have tried some of the various drugs prior to anesthesia, reputed to be specific, but my results have been very unsatisfactory. There is only one measure upon which I can depend with any certainty, and that is gastric lavage. In the majority of cases it will give a cessation of this symptom for a period varying from four to eight hours, when it may be repeated. A few drops of lemon juice added to a teaspoonful of water and administered at regular intervals will often have a quieting effect.

The paralysis that sometimes follows the administration of an anesthetic is generally due to faulty position of the patient or incorrect securing of the extremities, allowing undue pressure to be made on the nerve at some point along its course. A few cases are ascribed to the direct effect of an impure drug on the nerve proper.

As to the frequency of pneumonia, the Presbyterian Hospital of New York, from 1887-97, reports that 4914 ether cases were followed by seventeen pneumonias with nine deaths, and that 689 chloroform cases were followed by eight pneumonias with seven deaths. Schultze believes that this difference is due to the greater number of cases of malignant diseases of the mouth and respiratory tract among the chloroform subjects.

In conclusion, I recognize the fact that the important question of shock has been but lightly touched upon, and many points necessarily omitted as not bearing strictly upon the subject title.

